# *Streptococcus Thermophilus* UASt-09 Upregulates Goblet Cell Activity in Colonic Epithelial Cells to a Greater Degree than other Probiotic Strains

**DOI:** 10.3390/microorganisms8111758

**Published:** 2020-11-09

**Authors:** Madhur D. Shastri, Wai Chin Chong, Ravichandra Vemuri, Christopher J. Martoni, Santosh Adhikari, Harinder Bhullar, Dale Kunde, Stephen G. Tristram, Rajaraman D. Eri

**Affiliations:** 1 School of Health Sciences, College of Health and Medicine, University of Tasmania, Launceston 7250, Australia; ravichandra.vemuri@utas.edu.au (R.V.); sa3@utas.edu.au (S.A.); hbhullar@utas.edu.au (H.B.); dale.kunde@utas.edu.au (D.K.); stephen.tristram@utas.edu.au (S.G.T.); 2 School of Pharmacy and Pharmacology, University of Tasmania, Hobart 7005, Australia; 3 Department of Molecular and Translational Science, Monash University, Clayton 3800, Australia; waichin.chong@hudson.org.au; 4 Department of Pathology, Section of Comparative Medicine, Wake Forest School of Medicine, Medical Center Boulevard, Winston-Salem, NC 27157, USA; 5 UAS Laboratories, Madison, Windsor, WI 53598, USA; cmartoni@uaslabs.com

**Keywords:** probiotics, Mucin-2, goblet cells, intestinal peptides, immunomodulation

## Abstract

Probiotics have been widely used in maintaining gastrointestinal health, despite their actual mechanism remaining obscure. There are several hypotheses behind the beneficial effects of probiotics including the regulation of intestinal barrier function and improvement in immune responses in the gastrointestinal system. Multiple probiotics have been introduced in the market as effective dietary supplements in improving gastrointestinal integrity, but there are no or few studies that demonstrate their underlying mechanism. In the current study, we investigated and compared the efficacy of four probiotics (based on different bacterial species) in refining gastrointestinal health by improving mucus biosynthesis and intestinal immune response under in-vitro conditions. By analyzing the gene expression of mucus biosynthesis and intestinal immune response markers, we found that probiotic *Streptococcus thermophilus* UASt-09 showed promising potential in refining mucosal barrier and gastrointestinal health in human colonic epithelial cells, as compared to other commercial probiotics.

## 1. Introduction

Microbiota are found in many parts of the human body, of which the gastrointestinal tract contains the largest and most diverse collection [1]. It plays a pivotal role in digestion, nutrient absorption and removal of waste and microbial products. In addition to the microbial population, the gastrointestinal tract contains various cells carrying out individual roles [1]. These include enterocytes that absorb nutrients, Paneth cells secreting antimicrobial proteins, enteroendocrine cells involved in the production of multiple gastrointestinal hormones and microfold cells that sample pathogen antigens for intestinal immune system, while goblet cells produce mucin glycoprotein to form the outermost mucus layer on intestinal epithelial cells [2]. The gastrointestinal tract contains over 1000 microbial species identified so far and is constantly challenged by a myriad of commensal and/or pathogenic microbes daily [2]. As a protective measure, goblet cells produce a layer of mucin glycoprotein that act as a first line defense against harmful pathogens reaching the intestinal epithelial cells [3]. The mucus consists of 2 distinct layers: the outer loose layer (~100 um thick) that can be easily removed by gently suctioning, and the inner adherent layer (~50 um thick) that can be removed only by scraping [4]. The outer layer is comprised of attachment sites for microbes and nutrients that favor the colonization and growth of intestinal commensal flora [5]. Whereas, the inner mucus layer acts as a filter to separate the bacteria on the outer layer from invading into the intestinal epithelial cells [5,6].

Mucin glycoproteins are highly glycosylated and rich in serine and threonine [7]. So far, there are more than 20 different types of mucin glycoproteins that have been identified and named from mucin (MUC)-1 to MUC20 according to the order of their discovery [8]. Among them, MUC2 is the major secretory mucin glycoprotein produced by the goblet cells with a molecular mass of 540 kDa and consisting of 5179 amino acids [8]. It has a highly glycosylated central tandem repeat domain rich in proline, serine and threonine, allowing the monomer to get linked by other proteins having a cysteine-rich domain, such as von Willebrand factor [9]. With such characteristics, MUC2 mucin can easily undergo dimerization or oligomerization with other proteins, hence creating a gel-like semi-permeable network [10]. However, this mucosal barrier integrity is affected by the quantity and quality of the mucin glycoprotein produced by goblet cells and highly influenced by internal factors including bile salts, microbial by-products, hormones, and cytokines [11]. Besides MUC2 mucin glycoproteins, intestinal goblet cells also produce a myriad of intestinal peptides including Trefoil Factor 3 (TFF3), Resistin-like Molecule β (RELMβ), Fc-Gamma Binding Protein (FCGBP), and Anterior Gradient Homolog 2 (AGR2), and their specific functions are summarized in Table 1.

One of the common disorders involving the malfunction of the mucus layer is inflammatory bowel disease (IBD) [16]. IBD is an idiopathic inflammatory disorder, where the intestinal tract undergoes chronic or relapsing inflammation, leading to either ulcerative colitis or Crohn’s disease. While the actual mechanism behind IBD remains obscure, increasing studies have highlighted the involvement of gut microbiota in the pathophysiology of IBD [17,18,19,20,21,22]. For instance, Nishino and colleagues reported elevated levels of Proteobacteria like *Escherichia*, *Ruminococcus*, and *Cetobacterium* and reduction in *Firmicutes* and *Bacteroidetes*, such as genera *Faecalibacterium* and *Roseburia*, in endoscopic samples of patients diagnosed with IBD compared to a non-IBD control population [17]. Moreover, recent studies revealed that IBD patients have relatively lower spatial microbiota heterogeneity compared to healthy individuals [19,22]. Therefore, studies recommend the use of probiotics as a counter-measure for the restoration of gut microbiota/homeostasis in order to alleviate the risk of IBD [23,24,25]. It has been proposed that probiotics can restore the intestinal tract homeostasis either by disrupting pathogen signaling via the inhibition of quorum sensing [26], production of antimicrobial compounds that can inhibit pathogens [27], improving mucosal integrity against pathogens [28], or by competing for and reducing the adherence site for pathogens [29]. In the human gastrointestinal tract, *Lactobacillus*, *Bifidobacterium*, and *Streptococcus* subsp. have been widely trialed as probiotics in relieving diarrhea, irritable bowel syndrome, and IBD [30,31,32]. While there are many studies that reported the beneficial effects of probiotics and their contribution to longevity and digestive health, there are few studies that directly evaluate the efficacy of different probiotic strains in MUC2 mucin biosynthesis.

The current study tested multiple probiotic strains (obtained from diverse origins) and aimed to evaluate their efficacy in promoting gut mucosal health by assessing MUC2 mucin synthesis, increment in antimicrobial peptides such as TFF3, RELMβ, and other essential goblet cell-associated factors like AGR2 and FCGBP. Our study demonstrated that a *Streptococcus thermophilus* UASt-09 probiotic strain was superior to other tested probiotics in orchestrating a goblet cell activation program and a specific immune signature.

## 2. Materials and Methods

### 2.1. Bacterial Strains and Origins

Bacterial strains utilized in the study are summarized in Table 2. All other probiotic strains were obtained from UAS Laboratories, Windsor, WI, USA. The strains were cultured on De Man Rogosa agar supplemented with 0.05% (*w*/*v*) of L-cysteine (MRS-C), except UASt-09, which was cultured on M17 agar supplemented with 10% (*w*/*v*) lactose. All strains were incubated at 37 °C for 48 h under anaerobic conditions.

### 2.2. Cell Culture and Reagents

Human colonic epithelial cells LS174T were purchased from the American Type Culture Collection (ATCC, Manassas, VA, USA). For initial growth, the cells were cultured in 75 cm^2^ tissue culture flasks and grown in RPMI 1640 medium to reach confluence. The culture medium was supplemented with 10% fetal bovine serum (Sigma-Aldrich, St. Louis, MO, USA), 2 mM L-glutamine, and 100 U/mL of penicillin and streptomycin antibiotics and incubated at 37 °C in a humidified incubator containing 5% CO_2_. At confluence, the adhered cells were washed with PBS and dissociated with 0.1 *w*/*v* TrypLE^®^ Express (Gibco, Victoria, Australia). The cells were resuspended in 24-well cell culture plates (Corning, NY, USA) at a density of approximately 5 × 10^4^ cells/mL.

### 2.3. Treatments

The LS174T cells were incubated for 24 h and grown to confluency before exposing them to different treatments for the quantitative measurements of MUC2, TFF3, RELMβ, FCGBP, and AGR2. After 24 h, the supernatant media was discarded and replaced with serum-free media containing 10^9^ CFU/mL probiotic suspension and incubated again for 8 h. Following are the amounts of probiotics used to obtain a final concentration of 10^9^ bacterial cells/mL: *Bifidobacterium *animalis** subsp. *lactis* UABla-12 (15 mg/mL), *Lactobacillus acidophilus* DDS-1 (10 mg/mL), *Lactobacillus plantarum* UALp-05 (12 mg/mL), and *Streptococcus thermophilus* UASt-09 (20 mg/mL).

### 2.4. Reverse Transcription Quantitative Polymerase Chain Reaction (RT-qPCR)

Total RNA was isolated from cells by using the RNeasy Mini Kit (Qiagen, Hilden, Germany) following the manufacturer’s instructions. The concentration and quantity of RNA was determined using Experion™ Automated Electrophoresis System (Bio-Rad Laboratories, Hercules, CA, USA). The synthesis of cDNA was facilitated by reverse transcription of RNA using the iScript cDNA Synthesis Kit (Bio-Rad Laboratories, Hercules, CA, USA). The reaction conditions were: 5 min priming at 25 °C, 20 min for reverse transcription (RT) at 46 °C, 1 min for RT inactivation at 95 °C, and hold at 4 °C. The cDNA obtained were stored at −20 °C and utilized for reverse transcription quantitative polymerase chain reaction (RT-qPCR) and Prime PCR assay.

The RT-qPCR was performed on a Step-One Analyzer (Applied biosystem, USA) using a TaqMan Universal PCR Master mix (Applied biosystem, USA) with gene-specific primers for human MUC2, TFF3, RELMβ, FCGBP, and AGR2. The primer(s) information is provided in the Supplementary Data (Table S1). Briefly, 20 µL of PCR mix reaction volume was prepared by mixing 10 µL of TaqMan Universal PCR Master Mix, 1 µL of each forward and reverse primers of target gene, 1 µL of cDNA sample, and 7 µL of Nuclease-free water. The PCR conditions were as follows: 1 cycle at 95 °C for 2 min, 40 cycles of denaturation and extension phase at 95 °C for 5 s, then at 60 °C for 30 s and hold at 4 °C. The expression of the target genes was normalized to housekeeping gene Glyceraldehyde-3-Phosphate Dehydrogenase (GAPDH) and statistical analysis was performed.

### 2.5. Prime PCR Assay

The Prime PCR assay was also performed using the Step-One Analyzer (Applied biosystem, Foster, CA, USA). Unlike RT-qPCR, the gene-specific primers were pre-designed and pre-casted in each well of the purchased Prime PCR pre-designed Inflammation T1 H96 plate (Bio-Rad Laboratories, Hercules, CA, USA). Briefly, 20 µL of PCR mix reaction volume was prepared by mixing 10 µL of 2× SSo Advanced Universal SyberGreen Supermix (Bio-Rad Laboratories, Hercules, CA, USA) with 1 µL of cDNA sample and 9 µL of Nuclease-free water. The PCR conditions were as follows: 1 activation cycle at 95 °C for 2 min, 40 cycles of denaturation and extension phase carried out at 95 °C for 5 s, then at 60 °C for 30 s and hold at 4 °C. The expression of the target genes was normalized to housekeeping gene GAPDH and statistical analysis was performed.

### 2.6. Statistical Analysis

The data collected from the treated colonic cells was expressed as the mean ± SEM and samples were collected and analyzed in triplicates for each experiment. For the RT-qPCR experiments, Friedman two-way analysis of variance (ANOVA) with Bonferroni multiple comparison test was performed. Whereas, for the Prime PCR assay, the statistical comparison between the groups were performed using the Kruskal–Wallis one-way ANOVA with Bonferroni multiple comparisons test for post-hoc comparison. All statistical analyses were performed using IBM SPSS Statistics software version 26.0 (IBM, Armonk, New York, NY, USA) and data having the *p*-value of <0.05 were considered statistically significant. Graphs were plotted using GraphPad Prism version 6.0 (GraphPad Software Inc, San Diego, CA, USA). Error bars are presented as mean ± SEM, whereas the level of significance is presented as * = *p* < 0.05, ** = *p* < 0.01, *** = *p* < 0.001.

### 2.7. Expression Heatmap Analysis

The expression heatmap was generated by the online application “heatmapper” (link: http://heatmapper.ca). The data is column-clustered according to the expression of MUC2, TFF3, RELMβ, AGR2, and FCGBP in human colonic cells treated with *Bifidobacterium animal*is subsp. lactis UABla-12, *Lactobacillus acidophilus* DDS-1, *Lactobacillus plantarum* UALp-05, and *Streptococcus thermophilus* UASt-09.

## 3. Results

### 3.1. All Tested Probiotics Significantly Upregulate MUC2 Expression

**Figure 1:**
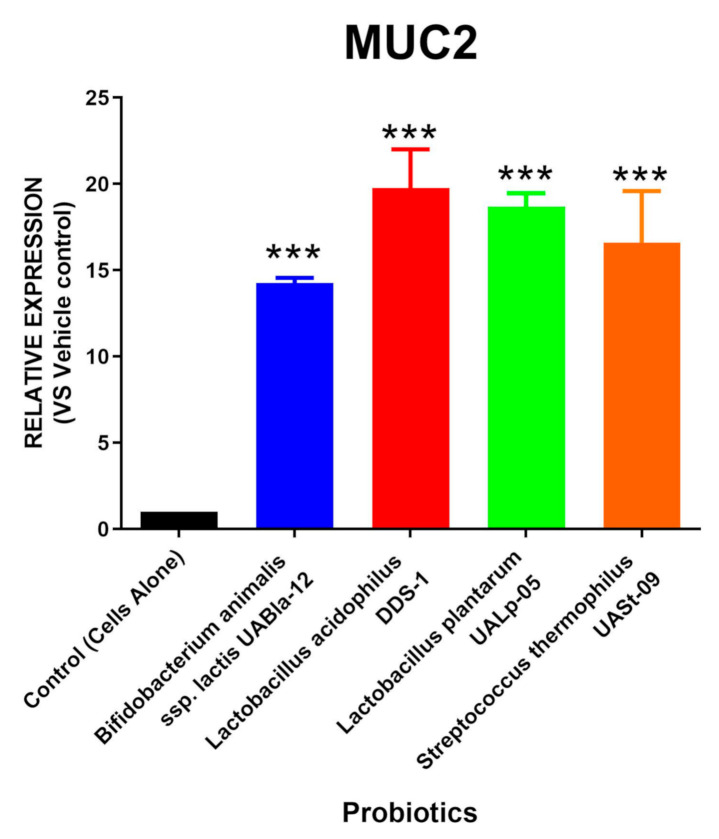
Probiotic-induced MUC2 production via colonic cells. LS174T colonic epithelial cells were grown to confluence and treated with multi-origin probiotics, namely, *Bifidobacterium animalis* subsp. *lactis* UABla-12, *Lactobacillus acidophilus* DDS-1, *Lactobacillus plantarum* UALp-05, or *Streptococcus thermophilus* UASt-09. The MUC2 gene expression after treatment (*n* = 3) was measured by real-time qPCR. Data is presented as mean ± SEM. *** p < 0.001.

In order to address the ability of MUC2 secretion in the tested human colonic cells, we treated the cells with four different probiotic strains (human, plant, and dairy origin, Table 2) and assessed the mRNA levels of MUC2 synthesis. The relative RNA expression of MUC2 levels were quantified before and after probiotic treatment and are shown in Figure 1. The expression of the MUC2 mucin was significantly elevated in the presence of probiotics, with *Lactobacillus acidophilus* DDS-1 having the highest expression (Mean = 19.775; SEM = 2.230) followed by *Lactobacillus plantarum* UALp-05 (Mean = 18.685; SEM = 0.775), *Bifidobacterium *animal**is subsp. *lactis* UABla-12 (Mean = 14.250; SEM = 0.310), and *Streptococcus thermophilus* UASt-09 (Mean = 16.597; SEM = 2.990).

### 3.2. Streptococcus Thermophilus UASt-09 Significantly Upregulates AGR2 Expression

**Figure 2:**
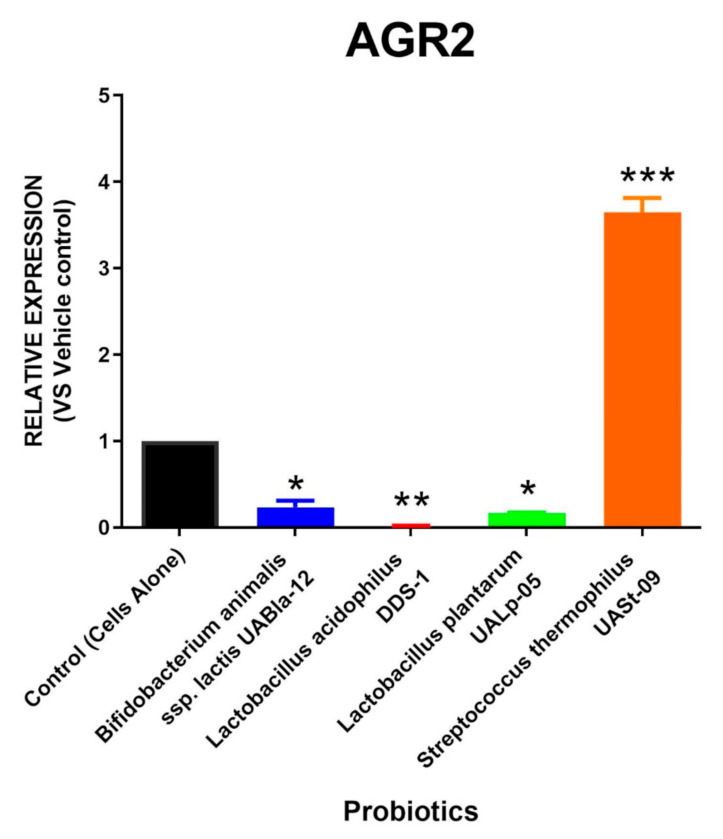
AGR2 release by probiotics. LS174T colonic epithelial cells were cultured and treated with *Bifidobacterium animalis* subsp. *lactis* UABla-12, *Lactobacillus acidophilus* DDS-1, *Lactobacillus plantarum* UALp-05, or *Streptococcus thermophilus* UASt-09. The expression of the AGR2 gene after the probiotic(s) treatment (*n* = 3) was measured by real-time qPCR. Data is presented as mean ± SEM. * *p* < 0.05, ** *p* < 0.01, and *** *p* < 0.001.

The probiotic *Streptococcus thermophilus* UASt-09 was found to significantly promote the expression of AGR2 (Mean = 3.648; SEM = 0.164), as shown in Figure 2. In contrast, other probiotic strains, such as *Bifidobacterium animal*is subsp. *lactis* UABla-12 (Mean = 0.235; SEM = 0.075), *Lactobacillus acidophilus* DDS-1 (Mean = 0.019; SEM = 0.003), and *Lactobacillus plantarum* UALp-05 (Mean = 0.165; SEM = 0.005), were found to significantly reduce the levels of AGR2.

### 3.3. All Tested Probiotics Enhance FCGBP Expression

**Figure 3:**
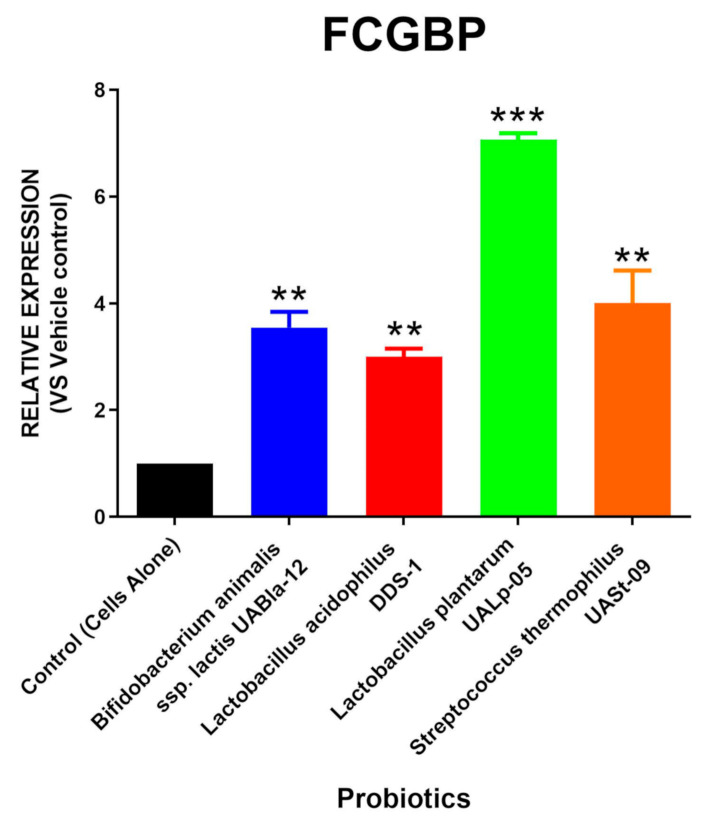
Probiotic-mediated FCGBP production. LS174T cells were grown under *in-vitro* conditions and treated with *Bifidobacterium animalis* subsp. *lactis* UABla-12, *Lactobacillus acidophilus* DDS-1, *Lactobacillus plantarum* UALp-05, or *Streptococcus thermophilus* UASt-09. The expression of the FCGBP gene after probiotic treatment (*n* = 3) was quantified via real-time qPCR. Data is presented as mean ± SEM. ** *p* < 0.01 and *** *p* < 0.001.

The relative RNA expression of FCGBP via the tested probiotic strains is shown in Figure 3. It was found that cells treated with *Bifidobacterium animal*is subsp. *lactis* UABla-12 (Mean = 3.550; SEM = 0.290), *Lactobacillus acidophilus* DDS-1 (Mean = 3.003; SEM = 0.150), *Lactobacillus plantarum* UALp-05 (Mean = 7.070; SEM = 0.120), and *Streptococcus thermophilus* UASt-09 (Mean = 4.006; SEM = 0.610) had a significant increase in the FCGBP expression compared to the untreated control.

### 3.4. Streptococcus Thermophilus UASt-09 Elevates RELMβ and TFF3 Expression

**Figure 4:**
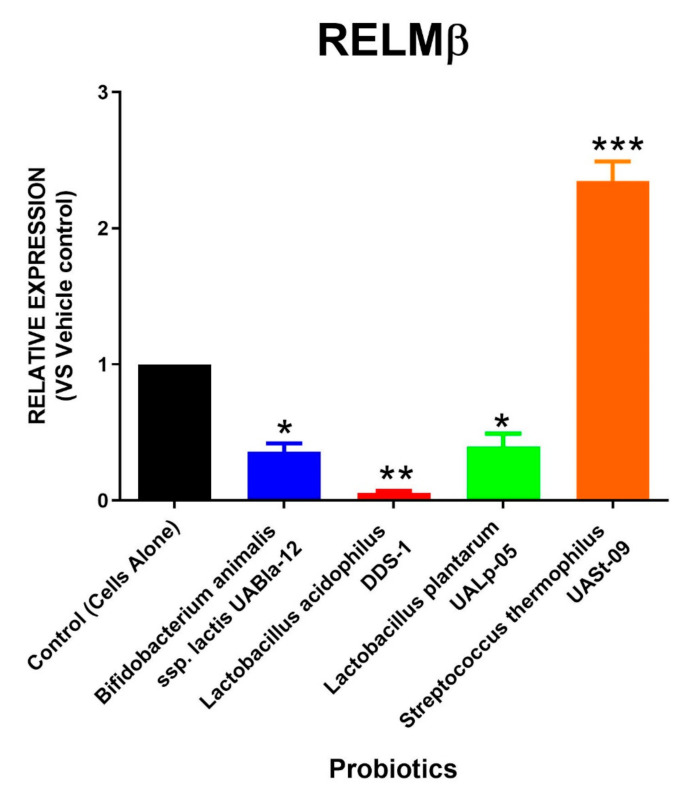
Expression of RELMβ in colonic cells via probiotics. LS174T colonic epithelial cells were cultured to confluence and treated with *Bifidobacterium animalis* subsp. *lactis* UABla-12, *Lactobacillus acidophilus* DDS-1, *Lactobacillus plantarum* UALp-05, or *Streptococcus thermophilus* UASt-09. RELMβ gene expression after probiotic treatment (*n* = 3) was assessed by real-time qPCR. Data is shown as mean ± SEM. * *p* < 0.05, ** *p* < 0.01, and *** *p* < 0.001.

**Figure 5:**
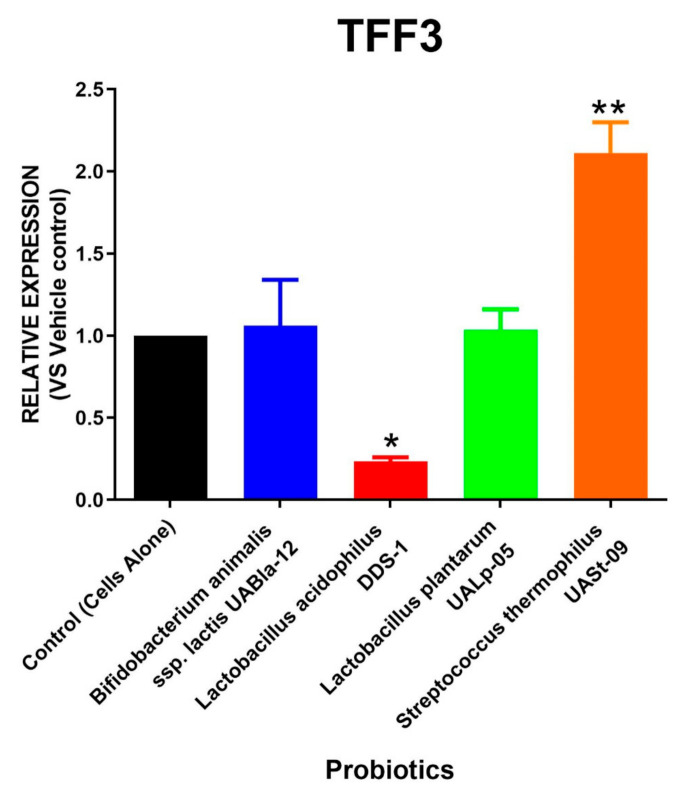
Production of TFF3 via colonic cells by probiotics. LS174T colonic epithelial cells were grown and challenged with *Bifidobacterium animalis* subsp. *lactis* UABla-12, *Lactobacillus acidop*hilus DDS-1, *Lactobacillus plantarum* UALp-05, or *Streptococcus thermophilus* UASt-09. The expression change in the TFF3 gene after probiotic treatment (*n* = 3) was computed by real-time qPCR. Data is presented as mean ± SEM. * *p* < 0.05 and ** *p* < 0.01.

Similar to AGR2 expression, the relative RNA expression of RELMβ and TFF3 after the treatment with different probiotic strains is depicted in Figure 4 and Figure 5. A single probiotic among the four tested strains displayed a significant upregulation in RELMβ as well as TFF3 levels. The cells treated with *Streptococcus thermophilus* UASt-09 had an approximately two-fold increase in expression of RELMβ and TFF3 (Mean = 2.345 and 2.114; SEM = 0.145 and 0.186, respectively). In contrast, *Lactobacillus acidophilus* DDS-1 was found to significantly downregulate the expression of both RELMβ and TFF3 (Mean = 0.055 and 0.236; SEM = 0.015 and 0.023, respectively). *Bifidobacterium animal*is subsp. *lactis* UABla-12 (Mean = 0.360; SEM = 0.060) and *Lactobacillus plantarum* UALp-05 (Mean = 0.395; SEM = 0.095) also helped downregulate RELMβ expression but did not alter the production of TFF3 compared to the vehicle control.

### 3.5. The Role of Streptococcus Thermophilus UASt-09 in Regulating Immune Response in Human Colonic Cells

**Figure 6:**
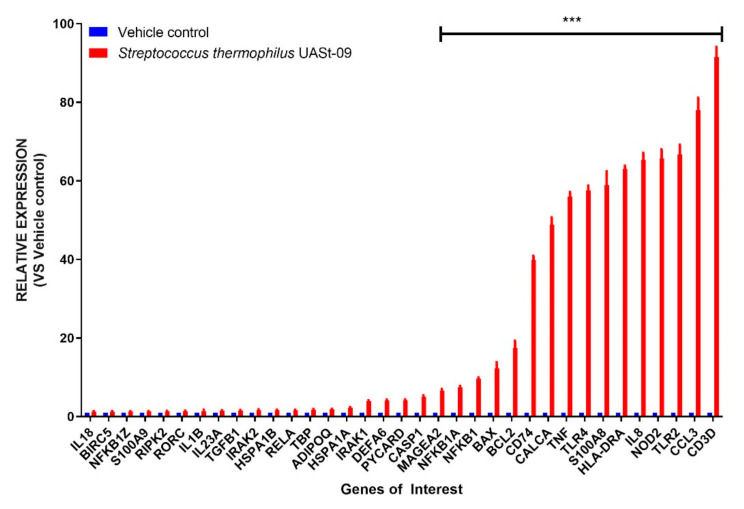
Prime-PCR Analysis. LS174T colonic epithelial cells were grown to confluence and treated with or without *Streptococcus thermophilus* UASt-09. The expression change of various genes after treatment was quantified by RT-PCR. Data is presented as mean ± SEM. *** *p* < 0.001.

Having observed the superiority of *Streptococcus thermophilus* UASt-09 in inducing goblet cell transcription, we further wanted to investigate its role in modulating immune responses in human colonic epithelial cells. To that end, we employed a multiplex immune/inflammatory panel for differential genomic expression of various immune markers. The expression of the tested immune regulators in human colonic cells after the treatment with *Streptococcus thermophilus* UASt-09 is summarized in Figure 6. Interestingly, the genomic expression of at least 16 immune markers was found to be significantly upregulated. Among them, the CD3D gene had the highest relative expression (Mean = 91.510; SEM = 2.583) followed by CCL3 (Mean = 78.007; 3.150), TLR2 (Mean = 66.655; SEM = 2.474), and NOD2 (Mean = 65.750; SEM = 2.291).

### 3.6. Heatmap Comparison of Probiotics

**Figure 7:**
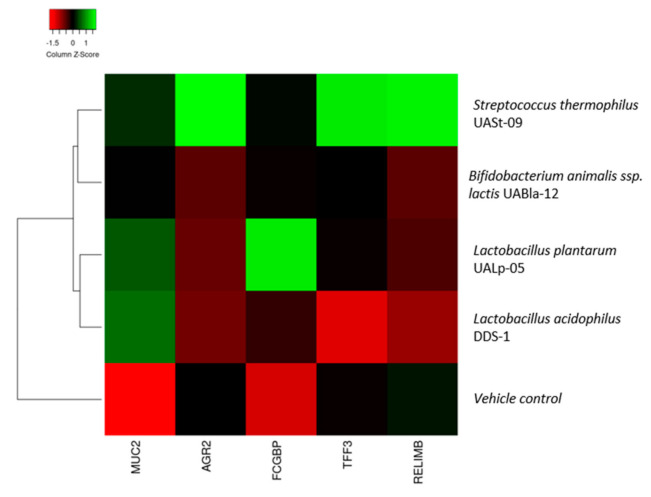
Heatmap comparison of *Bifidobacterium animalis* subsp. *lactis* UABla-12, *Lactobacillus acidophilus* DDS-1, *Lactobacillus plantarum* UALp-05, and *Streptococcus thermophilus* UASt-09 against vehicle control according to the MUC2, TFF3, RELMβ, AGR2, and FCGBP expressions via colonic epithelial cells treated with corresponding probiotics.

A heatmap comparison/summary is depicted, and as shown in Figure 7, there are 2 distinct categories of probiotics based on their treatment effect on colonic cells: strains that enhance both quality and quantity of mucin production and strains that only increase production of mucin. In this case, *Streptococcus thermophilus* UASt-09 is the only probiotic that exhibited improved quality and quantity of mucin production.

## 4. Discussion

Our investigation into the ability of multiple probiotic strains to regulate goblet cell function has demonstrated that *Streptococcus thermophilus* UASt-09 stands out as a goblet cell activator. It is possible that *Streptococcus thermophilus* has a metabolic advantage over the other tested probiotic strains because of its facultative nature, however, we believe this effect would be minimal for a number of reasons. Firstly, the levels of oxygen in the medium are generally relatively low, thus limiting the metabolic advantage. Additionally, the relatively short incubation time would limit bacterial replication and the very large initial inoculum would reduce the relative impact of the small number of replicative cycles that occurred. LS174T are specialized cells that resemble human goblet cells in appearance (contain mucus granules), secretion of major mucin (MUC2), antimicrobial peptides, and other essential goblet cell-associated factors, and possess a very similar transcriptional control mechanism [33]. The primary aim of the study was to assess goblet cell activation through several key molecules involved in transcriptional activity because the research into probiotic effects on increasing mucus production and goblet cell gene transcription is gaining momentum. The major component of human mucus is MUC2 protein whose transcription is tightly controlled by the goblet cell. Apart from MUC2 in the mucus (having a lubricating property), the protective ability against pathogens also comes from other antimicrobial peptides such as RELMβ and TFF3 [34,35,36]. In the current study, it was found that probiotics from diverse origins exhibit strain-specific effects on human colonic epithelial cells (that resemble goblet cells). All the tested probiotics were able to enhance MUC2 expression. MUC2 is essential for maintaining the intestinal homeostasis and is known to be critical for colonic protection [37,38]. Previously, MUC2^−/−^-deficient mice demonstrated increased permeability of the inner mucus layer resulting in bacterial invasion and inflammation [4,33]. Concurrently, MUC2 mutant mice (*Winnie*) develop spontaneous inflammation that mimic ulcerative colitis due to accumulation of non-functional mucin glycoprotein [39]. Additionally, *Winnie* mice exhibit aberrant immune responses resulting in chronic intestinal inflammation [40]. An increase in the expression of MUC2 via probiotics would in turn lead to an increased secretion of MUC2 granules into the intestinal lumen from the gastrointestinal goblet cells and help in strengthening the mucosal barrier integrity [41]. Similar to MUC2, cells treated with all the tested probiotic strains demonstrated an upregulation in FCGBP protein expression. FCGBP is an important component of mucin granules which forms disulfide bonds with TFF3 after covalent and/or non-covalent interaction and helps in the formation of a stable mucus layer [42]. Covalently bound FCGBP determines the structure and localization of the mucus layers in the gastrointestinal tract and, if there is a lack of FCGBP, mucus layers are no longer distinguishable [6,43].

However, only one probiotic strain, *Streptococcus thermophilus* UASt-09, significantly upregulated the expression of TFF3, RELMβ, and AGR2. TFF3 is the major secretory peptide of intestinal goblet cells and is involved in epithelial restitution and repair following mucosal injury [44,45]. The presence of intestinal TFF3 is known to interact with mucin and protects the intestinal epithelial mucosal layer against various harmful agents [45]. Moreover, in a study employing a mouse model deficient in TFF3, the administration of dextran sulfate sodium (DSS) resulted in severe colonic inflammation [44]. In addition, TFF3 also plays an essential role in protecting the mucosal barrier function and protection against the development of colonic inflammation [45]. RELMβ is a resistin-like molecule produced by the goblet cells and involved in divergent functions ranging from immune response following intestinal helminth infection, bactericidal action to limit the attachment of Gram-negative bacteria to intestinal epithelial surface and increased mucin secretion [14,46,47]. Mice deficient in RELMβ are more susceptible to bacterial infection, for instance, these mice demonstrated exaggerated mucosal damage and inflammation during *C*. *rodentium* infection [34]. Moreover, RELMβ-deficient mice were also found to have increased epithelial barrier permeability and susceptibility to chemical-induced colonic inflammation [48]. AGR2 is a disulfide isomerase located in the endoplasmic reticulum, responsible for the post-transcriptional regulation of MUC2 and is considered essential for its correct production [15]. Indeed AGR^−/−^ mice, whilst capable of producing MUC2, have a lesser developed, more permeable inner mucosal layer of the colon [49]. It is intriguing to note that only *Streptococcus thermophilus* UASt-09 helped induce the antimicrobial peptide transcription. While it is difficult to pinpoint a specific reason for the observed effects, the ability to specifically activate the antimicrobial peptides TFF3 and RELMβ appears to be unique and strain-specific. On the other hand, we observed that cells treated with *Bifidobacterium animalis* subsp. *lactis* UABla-12 and *Lactobacillus plantarum* UALp-05 showed significant downregulation in AGR2 and RELMβ levels. Similarly, *Lactobacillus acidophilus* DDS-1 treatment caused a significant reduction in TFF3 expression. Collectively, these results suggest that these particular probiotics can only promote the MUC2 mucin production, but are unable to maintain or improve the integrity of MUC2 mucin produced, and indicate that these strains might not be capable of strengthening mucus barrier function. Although speculative, the above observations indicate that the modulatory functions of these probiotics largely depend on the ability to specifically stimulate certain transcriptional pathways related to goblet cell function. In-depth (using deficient cell lines for these specific genes) mechanistic studies are warranted to decipher the regulation network of these different genes by probiotics within goblet cells.

This is the first study to have evaluated and compared the effects of probiotic strains obtained from different origins in the production of MUC2 glycoprotein and other essential peptides associated in maintaining mucosal integrity of the gut. We also addressed specific immune/inflammatory changes attributed to *Streptococcus thermophilus* UASt-09 as it was found to exhibit potential with respect to goblet cell activation. A number of immune markers where evaluated and a specific program that favored innate immunity was noted, namely activation of innate immune receptors or pattern recognition receptors (PRRs) such as TLR2, TLR4, and NOD2 were observed. Further, specific pro-inflammatory adaptive immune system markers such as IL18, IL23, and IL1β were considerably downregulated, demonstrating a modulatory effect of *Streptococcus thermophilus* UASt-09 in the gut mucosal system. Our study is well supported by a number of previous reports showing that probiotics help in mucosal tolerance by downregulating pro-inflammatory factor(s) [31,50,51]. While our prime PCR data for immune/inflammatory system provides a generalized map (difficult to pinpoint specific functions), detailed studies are warranted to investigate the immunological alterations assigned to specific probiotics. In general, the observed data obtained through *Streptococcus thermophilus* UASt-09 point towards a generalized upregulation of a number of genes/receptors that would be involved in infection/disease control. In other words, the preparatory stage of the colonic epithelial cells to ward off impending infection is perhaps orchestrated by the probiotics.

Moreover, it is difficult to link the observed effect of the probiotics to health and disease condition, and it is important to note that each probiotic can provide a different kind of beneficial effect by engaging in multiple signaling and activation pathways. In our study, *Streptococcus thermophilus* UASt-09 has the ability to induce the goblet cell activity which could be translated into conditions such as ulcerative colitis where the goblet cell is dysfunctional, leading to reduced mucus layer. Utilization of *Streptococcus thermophilus* UASt-09 may increase specific goblet cell activity in such conditions.

Limitations of the study include the lack of demonstrated protein expression data in relation to the gene transcription identified by qPCR. However, a number of studies show a direct correlation between gene and protein expression patterns for the genes investigated in our research work under similar conditions in LS174T or its derivative cell line [52,53].

## 5. Conclusions

We have demonstrated the potential functions of different probiotic strains on goblet cell-associated components in a species- and strain-dependent manner. In prior studies, the increased expression of MUC2, TFF3, FCGBP, RELMβ, and AGR2 have shown to strengthen and maintain the mucosal barrier integrity and gut protection. The current study was undertaken to identify a particular probiotic candidate that could elevate the specific goblet cell-associated factors. Of note, *Streptococcus thermophilus* UASt-09 was found to upregulate the expression of all components (MUC2, TFF3, FCGBP, RELMβ, and AGR2) of goblet cell activity. The present study highlights the potential role of *Streptococcus thermophilus* UASt-09 in modulating the mucosal barrier integrity and would be of interest to assess in future *in-vivo* and human clinical studies.

## Figures and Tables

**Table 1:** Goblet cell-secreted components and their specific roles in colon physiology.

Goblet Cell Secreted Components	Functions	Reference
Mucin 2 (MUC2)	Major component involved in the formation of mucus layer that acts as the first host-defense barrier	[4]
Fc-Gamma Binding Protein (FCGBP)	Stabilization and crosslinking of the MUC2 mucin barrier to the inner mucin layer	[6]
Trefoil Factor 3 (TFF3)	Associated in mucosal protection and healing process	[12]
Resistin-like Molecule β (RELMβ)	Involved in mucosal immune defense against microbial infection.	[13]
	Promote spatial segregation of the microbiota and the colonic epithelium	[14]
Anterior Gradient Homolog 2 (AGR2)	Essential in mucin biosynthesis	[15]

**Table 2:** Probiotic strains and species used in the study.

Genus	Species	Strain	Origin
*Bifidobacterium*	*animalis* subsp*. lactis*	UABla-12	Human
*Lactobacillus*	*acidophilus*	DDS-1	Human
*Lactobacillus*	*plantarum*	UALp-05	Plant
*Streptococcus*	*thermophilus*	UASt-09	Dairy

## Supplementary Materials

The following are available online at https://www.mdpi.com/2076-2607/8/11/1758/s1, Table S1: Information of the predesigned RT-PCR primers used in the study.
