# Supplementary material for: Streptococcus Thermophilus UASt-09 Upregulates Goblet Cell Activity in Colonic Epithelial Cells to a Greater Degree than other Probiotic Strains
Source: Microorganisms. 2020 Nov 9;8(11):1758. doi: 10.3390/microorganisms8111758 (PMC7695341; doi:10.3390/microorganisms8111758)
Supplement: Supplementary file 1 [file microorganisms-08-01758-s001.pdf]

Supplementary

Table S1. Information of the predesigned RT-PCR primers used in the study

Target Gene	Assay ID	Catalog Number	Supplier
Anterior Gradient Homolog 2(AGR2)	Hs00356521_m1	#4448892	Applied Biosystems
Fc-Gamma Binding Protein (FCGBP)	Hs00175398_m1	#4448892	Applied Biosystems
Trefoil Factor 3 (TFF3)	Hs00902278_m1	#4448892	Applied Biosystems
Resistin-Like Molecule β (RELMβ)	Hs00395669_m1	#4453320	Applied Biosystems
Mucin 2 (MUC2)	Hs03005103_g1	#4448892	Applied Biosystems
Glyceraldehyde-3-Phosphate Dehydrogenase (GAPDH)	Hs02786624_g1	#4331182	Applied Biosystems
